# Manganese–Cobalt Based Spinel Coatings Processed by Electrophoretic Deposition Method: The Influence of Sintering on Degradation Issues of Solid Oxide Cell Oxygen Electrodes at 750 °C

**DOI:** 10.3390/ma14143836

**Published:** 2021-07-09

**Authors:** Elisa Zanchi, Justyna Ignaczak, Bartosz Kamecki, Piotr Jasiński, Sebastian Molin, Aldo R. Boccaccini, Federico Smeacetto

**Affiliations:** 1 Department of Applied Science and Technology, Politecnico di Torino, Corso Duca degli Abruzzi 24, 10129 Torino, Italy; elisa.zanchi@polito.it; 2 Advanced Materials Centre, Faculty of Electronics, Telecommunications and Informatics, Gdańsk University of Technology, ul. G. Narutowicza 11/12, 80-233 Gdańsk, Poland; justyna.ignaczak@pg.edu.pl (J.I.); bartosz.kamecki@pg.edu.pl (B.K.); piotr.jasinski@pg.edu.pl (P.J.); sebastian.molin@pg.edu.pl (S.M.); 3 Advanced Materials Centre, Faculty of Applied Physics and Mathematics, Gdańsk University of Technology, ul. G. Narutowicza 11/12, 80-233 Gdańsk, Poland; 4 Department of Materials Science and Engineering, University of Erlangen-Nuremberg, Cauerstr. 6, 91058 Erlangen, Germany; aldo.boccaccini@fau.de

**Keywords:** electrophoretic deposition, manganese–cobalt spinel, solid oxide cell, chromium poisoning

## Abstract

This paper seeks to examine how the Mn–Co spinel interconnect coating microstructure can influence Cr contamination in an oxygen electrode of intermediate temperature solid oxide cells, at an operating temperature of 750 °C. A Mn–Co spinel coating is processed on Crofer 22 APU substrates by electrophoretic deposition, and subsequently sintered, following both the one-step and two-step sintering, in order to obtain significantly different densification levels. The electrochemical characterization is performed on anode-supported cells with an LSCF cathode. The cells were aged prior to the electrochemical characterization in contact with the spinel-coated Crofer 22 APU at 750 °C for 250 h. Current–voltage and impedance spectra of the cells were measured after the exposure with the interconnect. Post-mortem analysis of the interconnect and the cell was carried out, in order to assess the Cr retention capability of coatings with different microstructures.

## 1. Introduction

Volatile Cr species released from the steel interconnects of the solid oxide cell (SOC) stack, at different oxygen partial pressures, migrate and deposit on the air electrode, thus leading to a substantial and fast degradation of the electrochemical performance of the SOC cells. The outward Cr migration is partially limited by the introduction ~0.5 wt.% of Mn in the Crofer 22 APU alloy; anyway, the Cr–Mn spinel that develops at the top of the chromia scale has been reported to partially limit, but not completely avoid, the oxygen electrode degradation [1]. The application of a ceramic protective coating on the steel interconnects of SOC stacks is a widely employed solution to limit the steel oxidation rate and chromium evaporation [2]. In principle, the following two main factors influence the protective performance of such coatings: the chemical composition and densification. Among various proposed materials in the spinel family, perovskites and rare earth oxides, manganese–cobalt spinel coatings have shown a good balance in terms of their chromium blocking capability, matched thermal expansion coefficient, and high electrical conductivity at the SOC operating temperatures (500–850 °C) [3,4]. In recent years, research has been focusing on the implementation of the electrical and thermochemical properties of the base Mn–Co spinel, by transition metal doping (mainly Cu, Fe and Ni) [5,6,7], or rare earth element modification [8,9,10]. Different deposition methods have been reported in the literature for Mn–Co spinel coatings. For example, sputtering [11,12], electroplating [13], and plasma spray [14] allow highly dense coatings to be obtained, and a sintering treatment may not be required. On the other hand, when screen printing [15,16] or slurry-based methods, such as deep coating [17,18] and electrophoretic deposition (EPD) [19,20,21], are used, a post-deposition treatment is always required, in order to consolidate and densify the deposited powders and reduce the residual porosity. EPD is a fast and versatile process that allows homogeneous layers to be deposited in few seconds, and in a RT condition on complexly shaped steel components [22,23,24]. Furthermore, EPD offers the possibility to tune the spinel composition “in-situ”, by co-depositing the base spinel together with the desired amounts of Fe_2_O_3_ [25,26] or CuO [7,27], in order to obtain, respectively, iron or copper doped and modified manganese–cobalt spinel coatings. In a recently published article by Sabato et al. [28], EPD was proved to be an optimal deposition method to coat real dimension SOFC interconnects: despite the numerous corrugated and channeled surfaces on the Crofer 22 APU interconnect, the EPD-deposited Mn–Co spinel-based coating showed great stability and maintained protective properties after the stack test at 850 °C.

The coating deposition method and the consequent sintering profile are critical in obtaining well-performing protective coatings. Sintered coatings should be dense, in order to limit the possible chromium evaporation and gas access to the oxide scale. However, a certain degree of porosity can be considered beneficial, as this might stop possible cracks from propagating further. Although a two-step sintering process, consisting of a reduction followed by a re-oxidation step, is widely recognized as a valid post-deposition treatment to achieve high coating densification, it causes an increase in the processing cost of the interconnect [20,29]. Indeed, even poorly densified spinel coatings subjected to the one-step sintering in air could only ensure sufficient protection against Cr vaporization and corrosion of the interconnect during the stack operation at 800 °C [30]. In a previous study, Molin et al. [12] showed that the manganese–cobalt spinel coating that was deposited by EPD and submitted to the one-step sintering process (oxidation) in static air, reduced the oxide scale growth rate during the 5000 h tests at 800 °C, presenting the best protection in comparison to the manganese–cobalt spinel that was processed by physical vapour deposition methods. The study demonstrated the efficacy of a single-step sintering (thus excluding the reduction heat treatment step), in an effort to reduce the overall coating processing cost. This long-term study was focused on the area-specific resistance (ASR) degradation rate, while no indication of Cr poisoning of the cathode was reported. Moreover, with lower temperatures, as in IT-SOC, the relative importance of Cr evaporation compared to the oxide scale growth becomes larger [31]. The present study investigates the effect of coating density on cell performance and cathode poisoning at an operating temperature of 750 °C. To this purpose, a Mn_1.5_Co_1.5_O_4_ spinel coating was deposited on Crofer 22 APU substrates by the electrophoretic method. The deposited samples were then sintered following the one-step or two-step sintering processes, in order to obtain significantly different densification levels. Since the sintering procedure can play an important role in addressing the issue of preventing Cr evaporation, the final aim of this work is to evaluate the convenience of performing a two-step sintering process on the cell degradation rate for IT-SOC stacks. The results that are presented here show how different densification levels of spinel-based coatings can play a critical role on the reactivity with chromium, examining the relationship between the whole sintering procedure and the required manganese–cobalt coating performance.

## 2. Materials and Methods

### 2.1. Coating Deposition and Sintering

Steel coupons with a size of 15 × 15 mm^2^ were cut from a 0.3-mm-thick plate of Crofer 22 APU (Cr = 23 wt.%, Mn = 0.45 wt.%, La = 0.1 wt.%, Ti = 0.06 wt.%, Si and Al < 0.05 wt.%, Fe = Bal.) provided by VDM Metals (Verdohl, Germany). The coupons were cleaned in acetone and ethanol for 10 min each in an ultrasonic bath prior to deposition. The EPD suspension consisted of a mixture of ethanol and deionized water (60/40 vol.%) to which manganese–cobalt spinel powder with a chemical composition of Mn_1.5_Co_1.5_O_4_ (Fuelcellmaterials, d50 = 0.6 µm) was added to reach a solid content of 37.5 g/L. More details about the preparation of the suspension can be found in a previous work [32]. In order to coat both surfaces of the samples, electrophoretic deposition was performed in a three-electrode setup already described in [25], applying 50 V for 20 s and with a sample–electrode distance of 1 cm. After drying, the coated coupons were heat treated following two separate procedures. A first batch of samples was subjected to one-step sintering (oxidation only) at 900 °C for 2 h in static air; these samples are labeled 20s_Ox. On the other hand, the second set of samples were exposed to two-step sintering, consisting of a first heat treatment at 1000 °C for 2 h in flowing Ar/H_2_ 5% gas (reduction) and subsequently a second treatment at 900 °C for 2 h in static air (re-oxidation). These samples are therefore referred to as 20s_RedOx. Few samples were not subjected to the re-oxidation step to conduct morphological and compositional analysis of the coating after the reduction; these samples are labeled as 20s_Red.

### 2.2. Area-Specific Resistance Measurement Test

To examine the effect of the two sintering methods on the electrical properties, ASR measurements on the as-prepared 20s_Ox and 20s_RedOx coatings were performed. Platinum electrodes were painted (ESL 5542 conductive paste, ESL, Reading, UK) on both sides of the spinel-coated samples to form symmetrical electrodes in a cross-scale measurement configuration. The solvent from the paste was evaporated at 100 °C on a hot plate and then the samples were heated up to 750 °C for 10 min. The measurement was carried out in a four-electrode system with 5 mV sinusoidal amplitude of 1 Hz (Gamry Interface 1000 Potentiostat/Galvanostat/ZRA, Warminster, PA, USA). The sample was heated up to 750 °C, and then cooled to 200 °C with simultaneous impedance measurements. The ASR value of the samples was calculated, taking into account the electrode area, and divided by two to obtain a value for a single interface.

### 2.3. Interconnect Aging, Cr Evaporation Exposure and Fuel Cell Test

To evaluate the protective properties of the prepared coatings, aging test was performed. Anode-supported solid oxide fuel cells (ASC-SOFC, produced by TaipeiTech, Taipei, Taiwan) were used for the test. The 2.54 cm diameter cells were prepared by tape-casting/screen-printing processes. Structure of the cell consisted of Ni–YSZ porous support, Ni–YSZ active electrode, 25-μm-thick YSZ electrolyte, 8-μm-thick CGO diffusion barrier layer, and 30-µm-thick LSCF oxygen electrode with 1 cm^2^ active surface area. The cells have been exposed in the presence of differently coated steel interconnects in tube furnace, according to a procedure similar to the one reported in [21]. For cell performance evaluation the following three cells were aged: a reference cell without any steel placed onto the oxygen electrode, and two cells with coated steel described as 20s_Ox and 20s_RedOx, respectively. Each cell was aged separately to avoid possible Cr evaporation from the steel and poisoning mutually. The test was performed in a continuous flow of humidified atmospheric air for 250 h at 750 °C. In-between the exposures, the quartz tube was cleaned by an empty run with a chromium getter material. 

The cells after exposure were tested electrically for their fuel cell/electrolysis performance. For electrochemical performance, a Fiaxell Open Flange V5 test Set-up (Fiaxel Sarl, Lausanne, Switzerland) was used. The top side of the oxygen electrode was painted with La_0.6_Sr_0.4_CoO_3_ (Fiaxell, Lausanne, Switzerland) contact paste and dried before installation in the measurement setup. The electrical connection with a cell was provided by a gold grid from the cathode side and a Ni mesh on the anode side. The Au wires were connected to a Solartron 1260/1287 (Solartron Analytical, Leicester, UK) system for impedance and current–voltage characterization. Both electrodes were separated by alumina felt and working gases were directly supplied to the individual electrodes by ceramic tubes, i.e., avoiding any extra Cr poisoning source. The gas flows were controlled by mass flow controllers (Alicat Scientific, Tucson, AZ, USA). After heating to 750 °C, the reduction in the cell occurred in dry H_2_ until a stable OCV value (~1.1 V) was obtained. For cell performance characterization, current–voltage (I-V) characteristics and electrochemical impedance spectroscopy (EIS) were measured. The cells were examined in fuel cell mode using humidified H_2_ as a fuel and also measured under 50% H_2_O-50% H_2_ gas mixture for characterization in the electrolysis mode.

### 2.4. Morphological and Compositional Characterization

Morphological and compositional characterizations of both as-prepared and post-mortem samples were carried out by a field-emission scanning electron microscope (FESEM; SupraTM 40, Zeiss, Oberkochen, Germany) equipped with an energy dispersive X-ray analyzer (EDX, Bruker, Germany). To examine the cross sections, samples were embedded in epoxy resin and polished up to 4000 SiC paper. The coating’s porosity was evaluated by a graphical method using the ImageJ software (Version 1.53e, National Institutes of Health, Bethesda, MD, USA) [33], analyzing three SEM images from different regions of each sample.

## 3. Results and Discussion

### 3.1. Characterization of As-Prepared Samples

**Figure 1:**
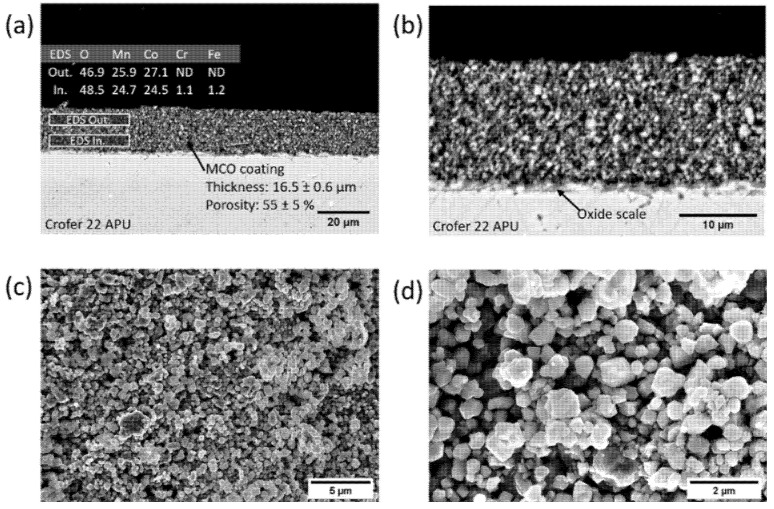
SEM images of 20s_Ox sample at different magnifications: (**a**,**b**) backscattered electron images and EDX analysis of the cross section; (**c**,**d**) secondary electron images of the top view.

Figure 1a,b shows the SEM images of the cross section of the coating submitted to the one-step sintering process. The coating is continuous throughout the sample surface, with a thickness ranging from around 16 to 17 µm. It is apparent from this image that the densification obtained after the oxidation treatment is poor, as confirmed by the calculated mean porosity, which exceeded 50%. However, the coating is very homogeneous, with the porosity being equally distributed over the whole thickness. It is evident that the coating adheres to the Crofer 22 APU substrate very well, and that there are no visible delamination phenomena or cracks at the coating/Crofer 22 APU interface.

The oxide scale developed during the heat treatment is visible, as the darker layer between the steel substrate and the coating, in Figure 1b. The irregular and jagged morphology prevents the accurate assessment of the thickness, which was found to be between 0.3 and 0.8 µm, which is in reasonable agreement with other studies [21,29]. The EDX analysis of the marked areas in Figure 1a demonstrates that the Cr concentration in the coating is almost negligible, despite being higher in the inner part.

The SEM images of the top view of the 20s_Ox sample are reported in Figure 1c,d; the MCO particles are still clearly distinguishable, proving that the heat treatment in air at 900 °C led to limited densification. In accordance with previous studies [12,32], it is evident that no phase transformation of the deposited spinel occurred as a result of the performed one-step sintering in oxidizing conditions.

**Figure 2:**
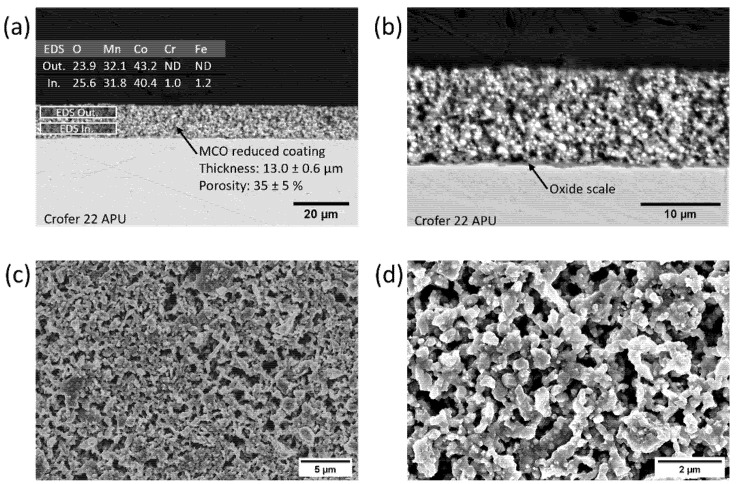
SEM images of 20s_Red sample at different magnifications: (**a**,**b**) backscattered electron images and EDX analysis of the cross section; (**c**,**d**) secondary electron images of the top view.

The SEM cross sections and top-view images of the 20s_Red coating and the related EDS elemental maps are shown in Figure 2a–d. In this case, the heat treatment in the reducing atmosphere at 1000 °C led to a higher coating densification. However, the residual porosity is still significant (approximately 35%). The performed heat treatment in the reducing atmosphere has already been proved to effectively cause the MCO powder to decompose into MnO and Co [25,34]. This is confirmed with the EDX area analysis reported in Figure 2a, as the relative amount of cobalt increases significantly. In particular, metallic cobalt particles correspond to the brightest spots in Figure 2b.

With an average thickness of 13 µm, the coating is continuous and well adherent to the steel substrate across the entire interface after the reducing treatment. The oxide scale is regular and presents good adhesion to the coating, thanks to the high-temperature treatment. Although the measured thicknesses lay in the same range as the 20s_Ox sample, the morphology of the chromia layer is very different among the two cases. Indeed, 20s_Red exhibits a compact layer, without inward protrusion in the steel (as observed in Figure 1b instead). Moreover, the Cr content in the coating is negligible, both in the inner and outer part (Figure 2a).The SEM top views of 20s_Red in Figure 2c,d reveal that the reduction was effective in producing partially densified structures, and the coating lost the appearance of packed particles, compared to the 20s_Ox sample. However, quite large porosities (≈1–2 µm) are present on the surface of the sample.

**Figure 3:**
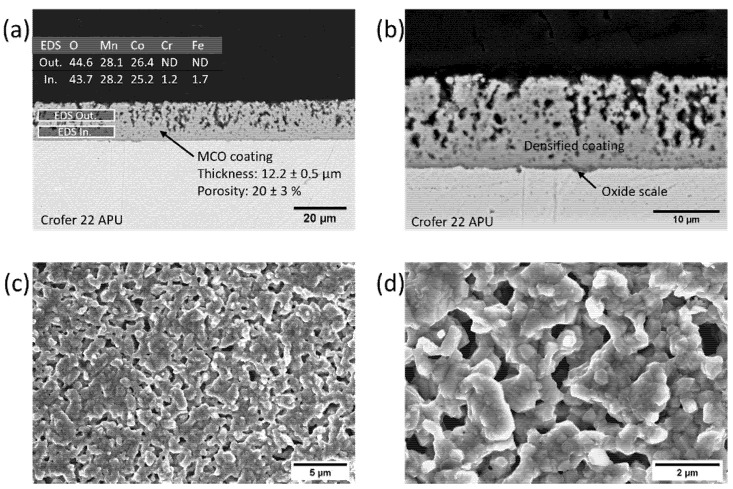
SEM images of 20s_RedOx sample at different magnifications: (**a**,**b**) backscattered electron images and EDX analysis of the cross section; (**c**,**d**) secondary electron images of the top view.

More interesting features can be appreciated by comparing the SEM images previously described, of 20s_Ox and 20s_Red, with those of the coating after the re-oxidation step (20s_RedOx), thus at the end of the two-step sintering process; the SEM cross sections and top-view images are presented in Figure 3a–d. During the re-oxidation step, MnO and Co are reported to react, in order to re-form the spinel structure and bringing benefits in terms of densification [20,25,29,32]. To this purpose, the most evident characteristic is the significant densification (80%) that is reached after the re-oxidation step, with a final coating thickness of around 12 µm (Figure 3a,b). Moreover, it is worth noting that the residual porosity is not homogeneously distributed throughout the coating. Indeed, a denser layer is easily distinguishable in the inner part of the 20s_RedOx coating (Figure 3b). This layer uniformly covers the oxide scale, thus protecting the steel from direct contact with air during operation of the device. Therefore, most of the porosity is concentrated in the outer part of the coating. Furthermore, the thickness of the oxide scale is very similar to the one measured after the reduction, even if few and small sub-scale nodules are distinguishable. They are formed because of the reaction of Cr, Mn and O in the oxidizing condition at high temperature [26]; however, their growth should be limited during long-term aging, thanks to the healing effect of the coating, which is completed after re-oxidation [25].

The EDX analysis reported in Figure 3a does not show any significant difference from what was already commented on for the previous samples, proving that the improved densification is not the consequence of any abnormal diffusion of neither Cr nor Fe from the steel during the heat treatment. Apparently, the metallic cobalt diffusion during the re-oxidation was very beneficial in densifying the partially sintered structures (already visible in the reduced samples in Figure 2c,d), closing or at least reducing the dimension of the porosity on the surface of the coating. The difference with the images presented in Figure 1c,d is obvious; here, the coating has completely lost the appearance of particles and the densified structures clearly exhibit the typical microstructure of the Mn–Co spinel.

**Figure 4:**
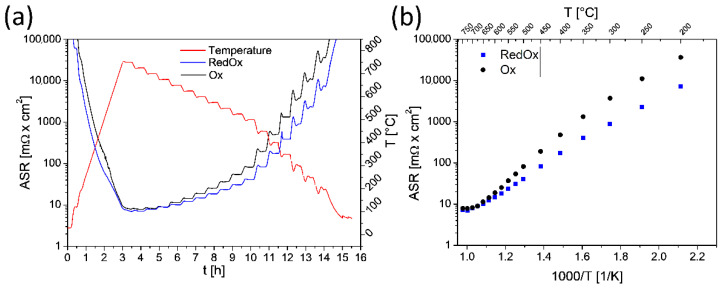
ASR measurement of the as-prepared 20s_Ox and 20s_RedOx samples: (**a**) as a function of time; (**b**) as a function of temperature.

To examine how the sintering process and coating density affect the electrical properties of the samples, the ASR values of the coated coupons were measured. The time and temperature dependence of the ASR is shown in Figure 4a,b, respectively. At 750 °C, the measured values are ~7.2 mΩ cm^2^ and ~7.9 mΩ cm^2^ for 20s_RedOx and 20s_Ox sample, respectively. As the temperature decreases, the difference in resistance increases, as follows: at 500 °C, the ASR measured for the 20s_RedOx sample is half that of the 20s_Ox sample (~40 mΩ cm^2^); at the lowest tested temperature, the ASR for the 20s_Ox sample is ~5 times higher than for the 20s_RedOX sample (~35 Ω cm^2^). The differences in these values are due to the different porosities of the deposited coatings, and the different thickness of the oxide scale.

### 3.2. Aging Exposure at 750 °C and Cell Test Results

**Figure 5:**
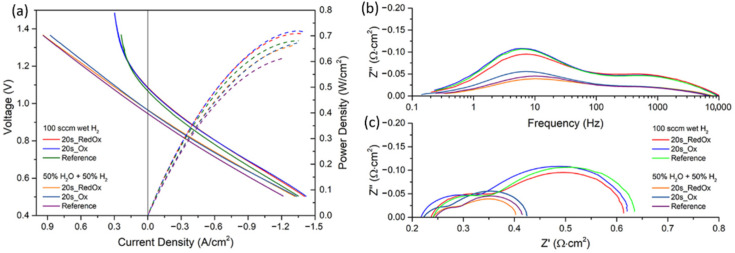
Plots of the examined cells measured at 750 °C: (**a**) I-V curves; (**b**) Bode plot; (**c**) Nyquist plot.

To evaluate the protective properties of prepared coatings, the electrochemical behavior of aged ASC-SOFCs was determined, using I-V and EIS characterization. Figure 5a–c, respectively, summarizes the current–voltage (I-V) and power density (PD) curves, and the impedance spectroscopy (EIS) results obtained at 750 °C under a continuous flow of synthetic air as the oxidant, and humidified hydrogen as the fuel. As shown in Figure 5a, the cells achieved an OCV of 1.07 V, and 1.09 V for the reference and both interconnect-exposed cells, respectively; moreover, the I-V curves and the maximum power densities were comparable for the cells exposed to the 20s_RedOx and 20s_Ox interconnects, i.e., achieved P_max_ of ~0.71–0.72 W/cm^2^, which is a typical performance of these cells. A minor difference was observed for the reference sample, which showed a P_max_ of ~0.68 W/cm^2^. A similar finding was observed for the cells that operated in the electrolysis mode, under 50% H_2_O-50% H_2_ as a fuel. The slope of all the I-V curves (reported in Figure 5a) was identical for voltages below the OCV, with an 0.02 V offset of the reference sample, possibly due to small leaks. Under the applied current load, the cell exposed to the 20s_RedOx sample presented a slightly better electrolysis performance.

The EIS measurement allowed the determination of polarization and ohmic resistances (R_pol_ and R_ohm_) at the OCV, and the corresponding Bode and Nyquist plots are shown in Figure 5b,c.

The minor differences are more than likely attributed to the reproducibility issues rather than to the effect of Cr evaporation and performance degradation.

As can be noticed on the Bode plot, the differences between the measured cells can be observed at a low-frequency range, between 100 Hz and 1 Hz. The process at 100 Hz–10 Hz can be related to the oxygen electrode process, while the processes at frequencies <10 Hz can be related to gas diffusion/conversion. For the 20s_RedOx sample, the mid-frequency process shows a slightly lower resistance in comparison to the 20s_Ox sample, both for the cells working at the fuel cell as well the electrolyzer mode, which could suggest poisoning of the oxygen electrode, even though Cr could not be detected in EDS.

For the cells working under humidified H_2_ as a fuel, the resistance values of the cells were similar. The cell that were exposed to the 20s_RedOx sample had R_ohm_ = 0.23 Ω∙cm^2^_,_ R_pol_ = 0.38 Ω∙cm^2^, the cell with the 20s_Ox sample had R_ohm_ = 0.22 Ω∙cm^2^ and R_pol_ = 0.40 Ω∙cm^2^, and the reference sample had R_ohm_ = 0.24 Ω∙cm^2^ and R_pol_ = 0.39 Ω∙cm^2^_._ There are no significant differences in the resistances of the compared cells in fuel cell mode. The situation is slightly different in the case of the cells tested in electrolyzer mode. While the reference cell and the cell aged with the sample 20s_RedOx have the same resistances of R_ohm_ = 0.24 Ω∙cm^2^ and R_pol_ = ~0.17 Ω∙cm^2^ values, the aged cell with the 20s_Ox coating shows slightly different values of R_ohm_ = 0.20 Ω∙cm^2^ and R_pol_ = 0.20 Ω∙cm^2^. The minor differences are more than likely attributed to the reproducibility issues rather than to the effect of Cr evaporation and performance degradation.

Interestingly, both the porous (20s_Ox) and dense (20s_RedOx) coatings are able to protect the cell from reaction with Cr vapors, in contrast with the work by Abdoli et al. [21]. In our case, the study was performed at a lower temperature (250 h at 750 °C instead of 100 h at 800 °C) and the cathode material was LSCF, whereas in [21] it was LSM. Also, the microstructure of the spinel coating differs: Abdoli et al. have used a thinner coating (≤5 µm), which can result in different evaporation rates.

To explain the protective behaviors of the 20s_Ox and 20s_RedOx coatings, microstructural characterization of the cells and interconnects was performed.

### 3.3. Post-Mortem Characterization

**Figure 6:**
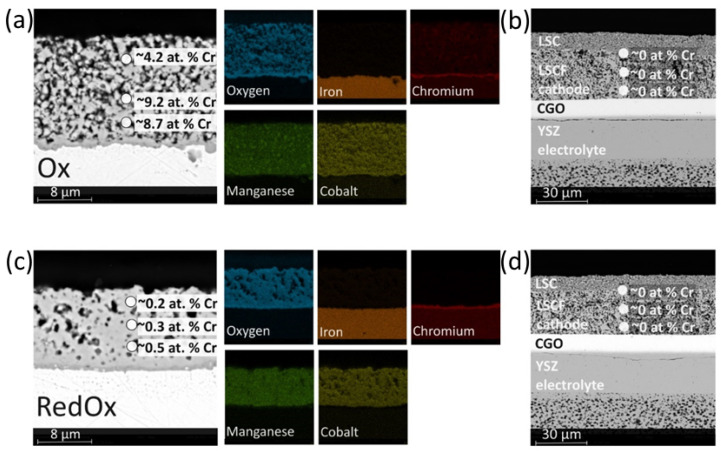
Post-mortem SEM analysis: (**a**) 20s_Ox sample; (**b**) fuel cell exposed to 20s_Ox sample; (**c**) 20s_RedOx sample; (**d**) fuel cell exposed to 20s_RedOx sample.

The microstructures and chemical compositions (elemental distribution) of the coatings, after 250 h of oxidation at 750 °C in humidified air flow, are shown in Figure 6a,c. Comparing the chromium distribution, large differences can be observed between the samples. For the two-step sintered 20s_RedOx sample, chromium only occurs at the steel–coating interface, i.e., forming chromium oxide scale. In the sample sintered only in air—20s_Ox—the chromium is present in the whole coating. According to the quantitative EDX point analysis, the amount of Cr in the 20s_RedOx sample varies from 0.5 at.% to 0.2 at.% above the chromium oxide layers and on top of the coating, respectively. For the sample with a higher porosity (20s_Ox), the chromium content is from 4 to 9 at.%, without following a concentration gradient from the steel to the top on the coating.

In addition, for the 20s_RedOx coating, the manganese-to-cobalt ratio is as expected (to a very good approximation 1:1), which is consistent with the stoichiometry of the raw spinel powder. The manganese content of the coating sintered in air (i.e. 20s_Ox) is higher than that of the starting powder, as the manganese-to-cobalt ratio is 1.13:1 (average obtained from five point EDX analyses), indicating Mn enrichment. This observation can indicate that a high-density coating slows down both chromium and manganese diffusion from the ferritic steel substrate.

The influence of the spinel coating microstructure (i.e., densification derived by different sintering treatments) on the Cr and Mn possible migration from the steel has already been discussed in various references. To this purpose, the importance of achieving a highly densified coating layer at the steel interface, to limit Cr and Mn diffusion, is reviewed in [20]. Moreover, a possible diffusion mechanism has already been proposed by Talic et al. [30], where the poorly densified oxidized spinel coating is shown to undergo slower, but continuous, densification at the steel interface during long-term aging. In [30], this has been evaluated by thermogravimetric measurement and Cr evaporation by the Denuder method. The long-term (3200 h at 750 °C) effect of the sintering temperature and atmosphere of the Mn–Co spinel-coated Crofer 22 APU has also been assessed in [26], in terms of the development of area-specific resistance over time. 

To identify if any chromium species can be found in the cathode, the same EDX analysis procedures as for the coatings was performed. The SEM images and point EDX analysis, which are presented in Figure 6b,d, were made on the LSCF cathode layer, which was placed directly above the coated steel (the visible LSC layer is the layer used for electrical measurement of the cell, which was applied after the high-temperature exposition). The point analysis of the LSCF cathode composition showed no chromium in either the 20s_RedOx or the 20s_Ox sample, which is consistent with the electrochemical results. The analysis of the EDX spectrum for LSCF is difficult to interpretate due to the overlap of the chromium and lanthanum peaks, but the qualitative comparison of the La and Cr signals does not indicate Cr diffusion. 

The study reveals that even the highly porous spinel coating reduced the Cr poisoning of the LSCF oxygen electrode. Though the porous coating is much more reactive than the dense one, for the relatively short test time and moderate temperature considered in this study, it seems to be a viable protective solution. In the porous 20s_Ox coating, the Mn–Co spinel reacts with the diffusing Cr and Mn, forming a mixed spinel, which must have a relatively low Cr evaporation rate, and acts as a Cr-getter material in this case.

## 4. Conclusions

High-quality manganese–cobalt spinel coatings, prepared by electrophoretic deposition, were evaluated for their potential to block Cr evaporation and poisoning of LSCF oxygen electrodes. Two different microstructures, including porous air-only and dense, redox sintered samples, showed noticeable differences in their Cr retention capability. Interestingly, both of the coatings were shown to block Cr evaporation and cell poisoning, as determined by the similar electrochemical performances (power density, ohmic and polarization resistance) of the cells after the aging exposures. However, microstructural post-mortem studies revealed the following large differences between the samples: the porous coating reacted with Cr and Mn diffusing from the Crofer 22 APU steel/oxide, whereas the dense 20s_RedOx coating remained mostly unchanged and virtually Cr-free. The porous spinel acted as a Cr-getter material, binding the Cr species and limiting further evaporation and cell degradation. The results show that the microstructure of the manganese–cobalt coatings has a large effect on their protective performance, and determines the possible mechanisms of degradation.

This study has shown that a highly dense coating can almost suppress Cr and Mn diffusion from the steel. The present investigation assesses the possible consequences on the electrode poisoning during the stack operation, by evaluating the Cr contamination of the oxygen electrode, finding that even the poorly densified coating (20s_Ox) can provide sufficient protection against electrode poisoning.

## Data Availability

Data is contained within the article.
